# Interfacial modification of basalt fiber filling composites with graphene oxide and polydopamine for enhanced mechanical and tribological properties

**DOI:** 10.1039/c8ra00106e

**Published:** 2018-03-29

**Authors:** Junjie Wang, Shaofeng Zhou, Jin Huang, Guizhe Zhao, Yaqing Liu

**Affiliations:** Shanxi Province Key Laboratory of Functional Nanocomposites, North University of China Taiyuan 030051 China zhoushaofeng@nuc.edu.cn lyq@nuc.edu.cn +86-351-3559669 +86-351-3559669; School of Chemistry and Chemical Engineering, Southwest University Chongqing 400715 China huangjin@iccas.ac.cn

## Abstract

Due to the chemical inertness of the basalt fiber (BF) surface, the weaker interfacial bonding between BF and polymer matrices will seriously affect the further application of basalt fiber enhanced composites. In this study, a continuous and compact graphene oxide (GO) layer was grafted onto the surface of basalt fiber (BF) using biomimetic polydopamine (PDA) as a bridge to improve the mechanical and tribological properties of polyamide 6. The impact and flexural strength of the PA6 composites filled by the GO grafting BF (GO–PDA–BF/PA6) indicated that the introduction of GO has made a larger improvement in interface bonding performance between BF and PA6 matrix. The friction and wear tests showed the wear rate of the GO–PDA–BF/PA6 composite decreased by 51% compared with BF/PA6 composites and it also showed the best wear resistance and load-carrying capacity under various applied loads and sliding speeds, explained by the improved interface bonding between GO–PDA–BF and PA6 matrix and the anti-wear protective transfer film formed by GO in the worn surface. This study provided a considerable flexibility strategy of tailoring the interfacial compatibility between reinforcement and matrix for effectively improving the comprehensive performance of composites.

## Abstract 2

Graphene oxide was grafted onto the surface of basalt fiber *via* polydopamine to enhance the interfacial adhesion of PA6 composites.

## Graphical abstract

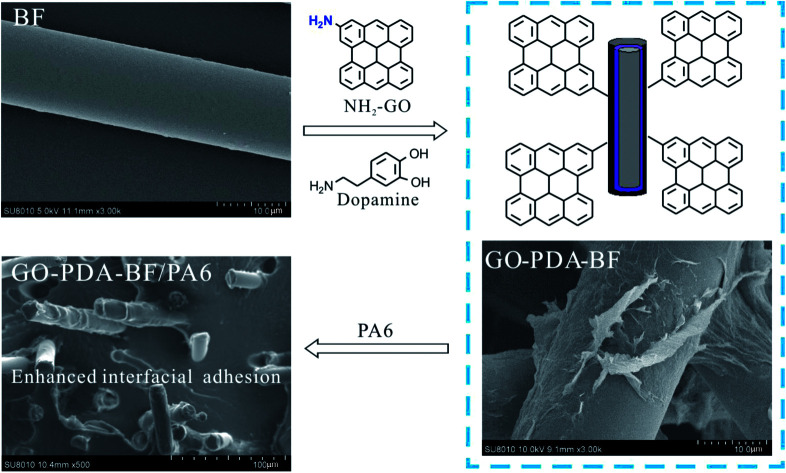

## 1. Introduction

As a reinforcement of composite materials, basalt fiber can substitute glass fiber and carbon fiber to a certain extent.^1–3^ But the poor interfacial adhesion between basalt fiber surfaces and matrix materials caused by the chemical inertness of basalt fibers cannot give full play to the advantages of basalt fiber and weakens its performance in composites. This severely inhibits their further application in every field. In the past few years, a lot of research work has been done to modify the surface of fibers to improve the interfacial interaction between fiber and polymer matrix, which to increase the mechanical and tribological properties of polymer composites.^4–8^ These works include surface treatments with various liquidus and atmospheric oxidation methods,^9–12^ electrochemical oxidation,^13,14^ plasma treatment,^15,16^ and fiber sizing or coating.^17–19^ For traditional chemical treatment methods, such as acid–base oxidation and electrochemical oxidation treatment, the enhancement of the chemical bond strength between the fiber and the matrix is achieved by increasing the active functional groups on the fiber surface. However, these treatments can damage the internal structure of the fiber to a certain extent, causing a certain degree of damage to the fiber. Compared with chemical oxidation treatment, the sizing or coating treatment of the fiber surface will not cause damage to the fiber simultaneously and the method is simple. However, considering the roughness of fiber surface could not be effectively improved, the degree of the chemical coupling and mechanical meshing between fiber and matrix are not satisfactory. Therefore, it is of great significance searching for a new method of without damage to the fiber and introducing a large number of active functional groups on the fiber surface to fiber reinforced composites.

For a long time, scientists have been studying the adhesion function of mussels, it is found that two hydroxyphenylalanine (DOPA) and lysine are the source of strong adhesion of mussels. On the basis of these findings, in 2007, dopamine, a substance with similar molecular structure with two hydroxyphenylalanine (DOPA), has become a new type of coating material that people focus on.^20–22^ The main advantage of dopamine is similar to that of mussel, it can polymerize and adhere to almost any substrates under a quite mild and facile condition, and the coating thickness is controllable and stable.^23,24^ As a result, the discovery of dopamine has opened up a new method of modifying the substrate and has caused extensive research. Dopamine is able to adhere to a large number of substrates through polymerization in a humid environment, and the polydopamine layer deposited with hydroxyl and imino groups can serve as a bridge for further functionalization.^25–27^ Therefore, the combination of biomimetic dopamine adhesion properties and nanomaterial modification will be a new method of surface modification.

Graphene oxide, with excellent intrinsic strength, fracture toughness and surface activity, has been employed as a promising mechanical strengthening component in various nanocomposites.^28–33^ Introducing graphene oxide onto the substrate of reinforcement could improve both the area, wettability of reinforcement, and lots of active functional groups could be modified on the surface of substrate, such as reinforcing fiber. Thus, grating GO onto the surface of fiber can been an interesting method to improve the interfacial stress between resin and fibers. Some studies found that the incorporating of GO on the surface of carbon fiber indicated 70.9% improvement of IFSS and 12.7% improvement of ILSS in comparison with the untreated carbon fiber/epoxy composites.^34^

In this work, polydopamine is generated by the polymerization of dopamine, which is coated on the surface of basalt fiber by its adhesion properties, and the graphene oxide was grafted onto the surface of basalt fiber with polydopamine as a bridge. The surface roughness and active group of basalt fiber were increased, and the mechanical bonding and chemical bonding between fiber and PA6 matrix were increased, so as to improve the interfacial bond between the fiber and PA6. The mechanical properties of basalt fiber enhanced PA6 composites were evaluated by impact and flexural tests. Furthermore, the tribological properties of the composites were systematically studied under different stress loads and sliding speeds. The results showed that the graphite oxide modified basalt fiber reinforced PA6 composite exhibits excellent mechanical properties and wear-resisting properties.

## 2. Experimental details

### 2.1. Materials

Short basalt fiber (BC13-2400W) with a mean diameter of 13 μm and a length of 200 μm were produced by Shanxi Jintou Basalt Development Co. Ltd (Shanxi, China). Polyamide 6 (PA6, 1013B) was obtained from Ube Industries (Japan). The graphene oxide was obtained from Tangshan Jianhua Technology Development Co., Ltd (Tangshan, China). 3-Hydroxyphenethylamine hydrochloride (dopamine, 98%) was also obtained from Wuhan GeAo Chemical Technology Co. Ltd (Wuhan, China). Tris-(hydroxy-methyl)aminomethane (Tris, 99%), which is used as a buffer agent, were purchased from Shanghai Mclean Biochemical Technology Co., Ltd (Shanghai, China). Ethylenediamine was obtained from Tianjin Fuchen Chemical Reagent Factory (Tianjin, China). All chemical reagents and solvents were used as received and without further purification.

### 2.2. Preparation process

#### 2.2.1. PDA coating of BFs

**Fig. 1:**
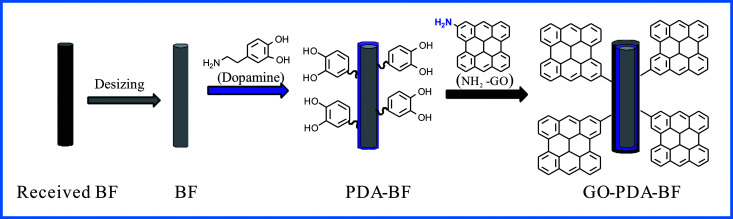
Process of graphene oxide grafting onto the surface of basalt fiber.

The as-received BFs were cleaned in soxhlet extractor at 80 °C for 48 h with acetone to remove the commercial sizing before being used, which were denoted as BF. To prepare buffer solution, the 40 mM TRIS was added to the 2000 mL deionized water, its pH was adjusted to 8.5 with hydrochloric acid, and 40 g BF was added to the buffer solution. Followed by the addition of 4.0 g dopamine, the mixture was ultrasonic treatment for 10 min and then stirred magnetically for 24 h at room temperature. At this reaction conditions, PDA were coated on the surface of BFs *via* pH-induced self-polymerization of dopamine. The PDA-coated BFs (PDA–BF) were collected *via* filtration, washing and freeze drying. The process of polydopamine coating and graphene oxide grafting onto the surface of basalt fiber was presented in Fig. 1.

#### 2.2.2. Amino functionalization of GO

1.0 g graphene oxide was treated with deionized water to form a GO solution at the concentration of 1.0 g L^−1^. Put ethylenediamine into the GO solution, then agitated the mixture magnetically at 60 °C for 12 h. Thereafter, extracted the reaction solution through centrifugation, and finally got the amino functionalization oxide grapheme (NH_2_–GO) by drying the solid product at 60 °C for 24 h.

#### 2.2.3. Grafting NH_2_–GO onto the surface of PDA-coated BFs

**Fig. 2:**
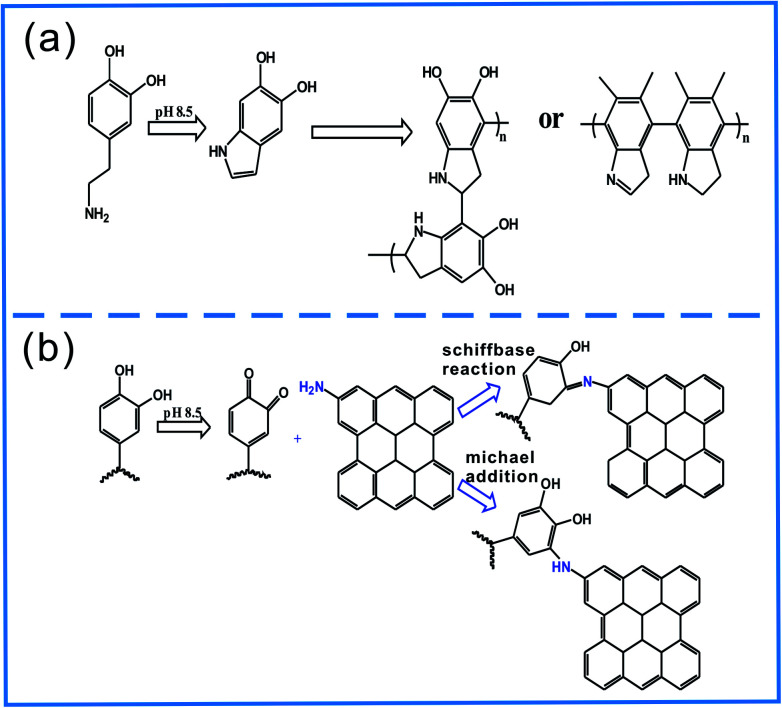
Reaction mechanism of polydopamine coating (a) and GO grafting (b).

Firstly, PDA–BF was immersed in the Tris buffer solution. Then added the NH_2_–GO into the solution and stirred it at room temperature for 24 h. Washing three times with deionized water after filtering to remove unreacted NH_2_–GO, the NH_2_–GO functionalized BFs (GO–PDA–BF) were obtained by drying the products at 60 °C under vacuum for 24 h. The possible chemical reactions in this process are shown in Fig. 2.

#### 2.2.4. Preparation of GO–PDA–BF/PA6 composites

The GO–PDA–BF were mixed with PA6 to prepare GOPDA–BF/PA6 composite samples using a TE-20 co-rotating twin-screw extruder (Nanjing Keya Coperion Machinery Co. Ltd., China) at a screw speed of 30 rpm and feed rate of 15 rpm. The temperature profile of the barrel was set at 225–230–235–240–230 °C from the hopper to the die. The composite is sheared into small particles by a granulator and dried in oven at 80 °C for 12 h before injection molding. Standard impact and flexural test bars were prepared respectively by injection mold using SZ-100/80 plastic-injection molding machine (Shanghai Plastic Machinery Factory, China). The injection temperature of each stage was 225–235–240–245–250 °C, respectively.

### 2.3. Characterization methods

X-ray Photoelectron Spectroscopy (XPS) analysis of the GO was performed using a Thermo ESCALAB 250Xi system X-ray photoelectron spectrometer. The scanning electron microscopy (SEM) images of the basalt fibers, fracture surface and worn surface of the composite were obtained on a Hitachi SU-8010 field emission SEM system (operated at 10 kV).

The flexural and impact properties of the PA6 composites were conducted on a AI-7000s universal electronic testing machine according to GB/T 9341-2008 and GB 1843-2008 (Chinese Standard).

The worn surfaces of the PA6 composites were examined using a three dimensional non-contact surface topography instrument and profile measurement apparatus with a repeated accuracy of 0.012 μm (ST400, NANOVEA, USA). The friction and wear tests were performed on a multifunctional material surface comprehensive performance tester (CFT-I, Lanzhou Zhongke Kaihua Technology Development Co., Ltd) under dry conditions. The dual samples bearing stainless ball diameter is 5 mm, as provided by the supplier. The friction coefficient (μ) was exported automatically *via* the computer dealing with collected data and the wear volume loss (Δ*V*, mm^3^) was obtained according to real wear tracks measured by a micrometer (resolution: 0.0001 mm). The wear rate (*K*, mm^3^ N^−1^ m^−1^) of the specimen was calculated from the following equation:*k* = Δ*V*/*FtL*where *F* is the applied load (N), *t* is the experimental duration (min), and *L* is the stroke length in one cycle (m). In this work, three readings of the friction and wear tests of steady-state sliding under dry conditions were taken, and the average values were adopted in our results.

## 3. Results and discussion

### 3.1. Structure characterization of GO grafting onto the surface of basalt fiber

In order to improve the grafting efficiency of GO onto the surface of BFs, GO were functionalized by amino and BFs were coated by PDA. The amino of GO–NH_2_ and hydroxyl of PDA coated BFs would produce the Schiffbase reaction and Michael addition reaction under alkalinous condition.

**Fig. 3:**
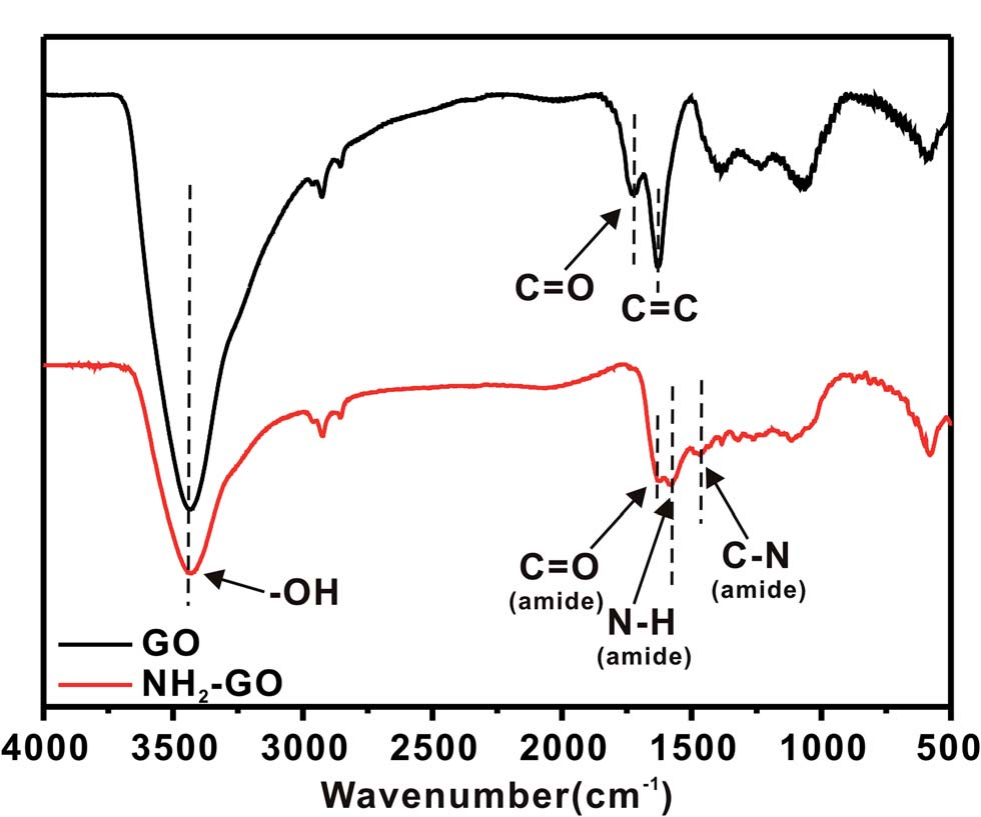
FT-IR spectra of GO and NH_2_–GO.

Fig. 3 shows the FT-IR spectroscopy results of GO and NH_2_–GO. The peaks located at 3430 cm^−1^,1724 cm^−1^ and 1630 cm^−1^ in the GO spectrum indicate the presence of –OH, C=O and C=C bonds, respectively, suggesting the existence of hydroxyl and carboxyl groups on the surface of GO. The spectra of the NH_2_–GO have several new peaks that are not present in the GO spectrum. The appearance of new peaks at 1640 cm^−1^, 1592 cm^−1^ and 1410 cm^−1^ in the spectra of the NH_2_–GO indicates the formation of C=O (amide), N–H and C–N, and the intensity of the carboxyl vibration absorption peak decreases, which is further indicated that the amino have been successfully modified to the surface of GO.

**Fig. 4:**
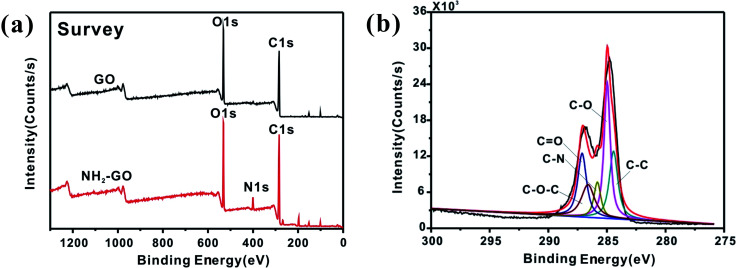
XPS wide-scan (a) and C 1s core-level spectra (b) of NH_2_–GO.

The XPS results proves that amino group has been successfully functionalized on GO. The XPS spectrum in Fig. 4a shows C, O and N elements could be seen from the diagram corresponding to the NH_2_–GO. Fig. 4b is a fitting result of the C 1s spectrum corresponding to NH_2_–GO, which consists of five peaks in 284.4 eV, 284.9 eV, 285.8 eV, 286.6 eV and 287.7 eV, corresponding to C–C, C–O, C–N, C–O–C and C=O,^35^ respectively. Table 1 listed the content of each element for NH_2_–GO and pure GO. It can be found that NH_2_–GO contains 5.9% of N element.

**Table 1:** Elemental analysis of pure and functionalized GO by XPS

	C (%)	N (%)	O (%)
GO	72.5	0	27.5
NH_2_–GO	70.6	5.9	23.5

**Fig. 5:**
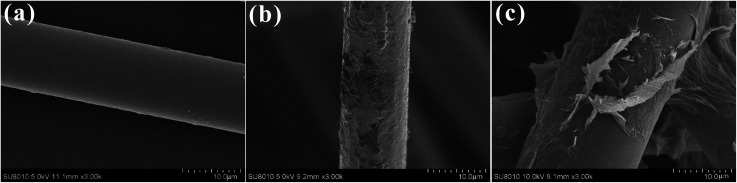
SEM images of the surfaces of BF (a), PDA–BF (b), and GO–PDA–BF (c).

The coating quality on the surface of BFs, in terms of homogeneity and dispersion of GO, was characterized by SEM. After removing the commercial coating, Fig. 5a shows the surfaces of pristine basalt fiber were very smooth with few pits and grooves. But after self-polymerization of dopamine on the surface of BFs, a thin and even layer was observed on the PDA–BF surfaces (see Fig. 5b). However, when the GO were grafted onto the surface of BFs, the surface morphology of basalt fiber has changed greatly as shown in Fig. 5c. From the chart, we can see that GO sheets has been successfully grafted onto basalt fiber surface to form a very stable structure, and the roughness of fiber surface has been obviously improved.

### 3.2. Mechanical properties of the BF/PA6 composites

**Fig. 6:**
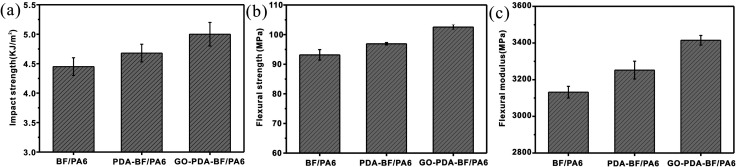
Impact strength (a), flexural strength (b) and flexural modulus (c) of BF/PA6, PDA–BF/PA6 and GO–PDA–BF/PA6 composites.

The impact and flexural tests were conducted to investigate the interfacial adhesion properties of the basalt fiber reinforcement PA6 composites. As given in Fig. 6, the impact strength of the PDA–BF/PA6 composite was increased by 6.4%, and the impact strength of GO–PDA–BF/PA6 composites achieved 13.6% improvement. Compared with BF/PA6 composite, the flexural strength and flexural modulus of GO–PDA–BF/PA6 composites achieved 12.7% and 11.1% improvement, respectively. The results showed the BF surface modification of GO and PDA would effectively improve the impact strength, flexural strength and flexural modulus of BF/PA6 composite. The forming GO sheets coating on the surface of BF would significantly increase the roughness of BF, which will improve the mechanical interlocking between BF and PA6 matrix.^36,37^ Besides, the more polar functional group of GO would offer more opportunity to increase the interaction between BF and molecular chain segments of PA6. On the whole, the interfacial adhesion of GO–PDA–BF/PA6 composites would benefited from both the chemical bonding and mechanical meshing between BF and PA6 matrix, in addition, because GO has larger area and thinner nanostructure, when composites are subjected to stress, they tend to deform and absorb more energy, and when the composites are cracked, GO can quickly fill the crack and inhibit the further expansion of the crack and play a reinforcing role, thus their mechanical properties were at the high lever.^38,39^

**Fig. 7:**
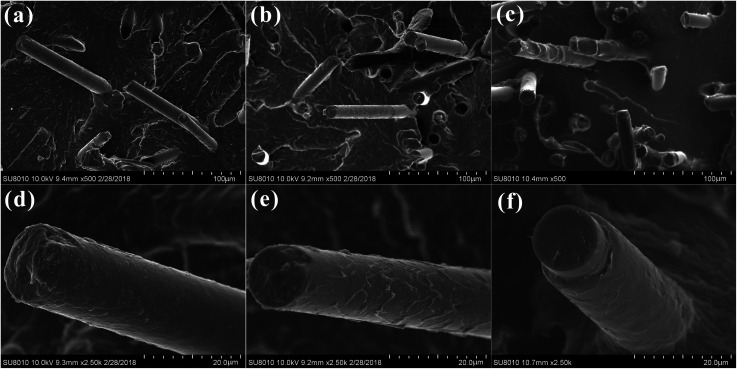
SEM micrographs of impact fractured surfaces for BF/PA6 (a), PDA–BF/PA6 (b), GO–PDA–BF/PA6 (c) and their corresponding magnified image in (d)–(f).

Fig. 7a–c displayed the SEM micrographs of impact fractured surfaces for the BF/PA6 composites, PDA–BF/PA6 composites and GO–PDA–BF/PA6 composites, respectively. The surface of the BF pulled out in the section is smooth, and the PA6 matrix without remains on the surface of the fiber in Fig. 7a and d, which is due to the poor interface binding property between BF and PA6 matrix. After coating with PDA, a small amount of matrix with a thin layer structure was left on the surface of the extracted PDA–BF as show in Fig. 7b and e, which is because the interfacial bonding strength between the fiber and PA6 matrix is improved. As to the GO–PDA–BF/PA6 composites, much thicker adhesive matrix was adhered to the GO–PDA–BF surface and few debonding phenomena could be found (Fig. 7c and f). By increasing the interaction between chemical bonding and mechanical meshing, the action stress can be effectively transferred into the fiber to suppress the formation and expansion of the crack. SEM observation shows that PDA and GO can effectively improve the interfacial bonding force between basalt fiber and polymer matrix.

### 3.3. Tribological properties

#### 3.3.1. Friction and wear behaviors of GO–PDA–BF/PA6 composites

**Fig. 8:**
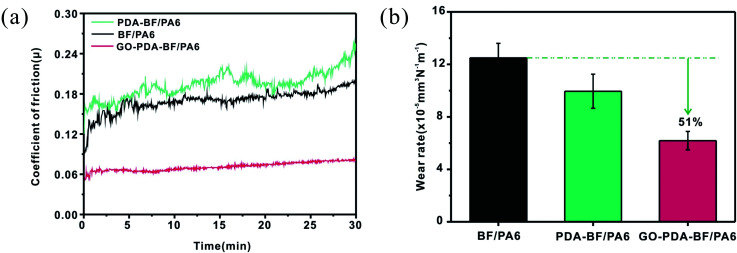
Friction coefficient (a) and wear rate (b) of BF/PA6, PDA–BF/PA6 and GO–PDA–BF/PA6 composites (70 N, 0.167 m s^−1^, 30 min).

Fig. 8 shows the influence of friction coefficient and wear rate of PA6 composite filled by GO grafting basalt fiber. Seen from Fig. 8, the friction coefficient of PDA–BF/PA6 composites is higher than that of BF/PA6 composites, while the wear rate is lower than that of the BF/PA6 composite materials. In the BF/PA6 composite, the surface of desizing BF is smooth and lacks polar groups, which causes the poor interface bonding between BF and nylon6 matrix. The cracks will be generated more easily between the fiber and the matrix during the friction process, and thus the composite will be loosened. In this condition, the corresponding friction force and friction coefficient will decline, and much more fiber will be detached from the matrix. Therefore, the wear rate of BF/PA6 was at a high level. After grafting GO onto the surface of BF, the friction coefficient and wear rate of GO–PDA–BF/PA6 composites reached the minimum value, and it is well worth noting that wear rate of the composite decreased by 51% when filled by GO–PDA–BF. This may be caused by the increased roughness and reactive functional groups of GO grafted on the surface of basalt fiber, which improves the interface bonding strength between BF and PA6 matrix. The improved interfacial adhesive will alleviate the debonding of BF, crack propagation and matrix damage on the worn surface. And during the friction process, GO will gradually appear on the surface of friction parts and play an effective role in friction reduction and lubrication. Therefore, together with the mechanical properties, it is reasonable to believe that the wear resistance of BF/PA6 composites can be improved obviously by grating GO on its surface of BF.

#### 3.3.2. Friction and wear behaviors under different loads

**Fig. 9:**
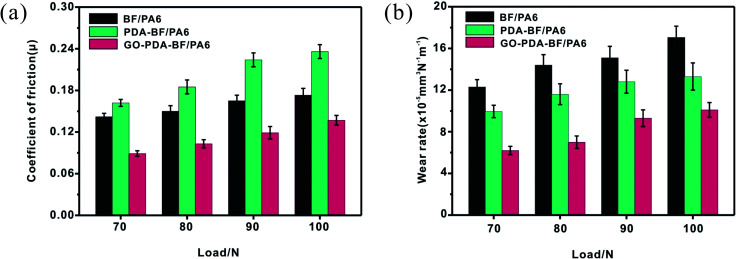
Friction coefficient (a) and wear rate (b) of BF/PA6, PDA–BF/PA6, and GO–PDA–BF/PA6 composites as a function of applied load (0.167 m s^−1^, 30 min).

Fig. 9 showed the results of wear tests under various applied load for BF/PA6, PDA–BF/PA6 and GO–PDA–BF/PA6 composites, respectively. It can be seen that the friction coefficients and wear rate of these three composites increased with the increasing of applied load. With the increase of load, the fiber and part of matrix are stripped from the composite, the roughness of the worn surface increases, and the crack is further expanded. Therefore, the friction coefficient and wear rate increase.^40^ For the GO–PDA–BF/PA6 composite, the improvement of interface bonding strength can effectively relieve stress concentration and inhibit the formation of cracks, which will significantly improve the wear resistance of composites.

#### 3.3.3. Friction and wear behaviors under different sliding speed

**Fig. 10:**
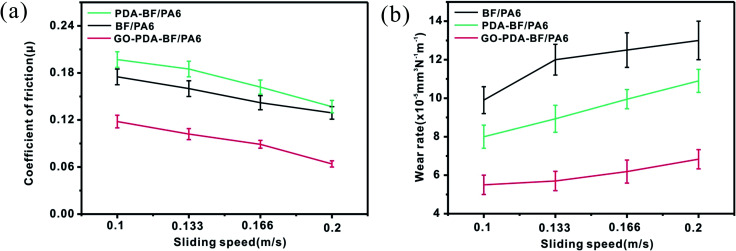
Friction coefficient (a) and wear rate (b) of BF/PA6, PDA–BF/PA6, and GO–PDA–BF/PA6 composites as a function of sliding speed (70 N, 30 min).

Fig. 10 studies the effect of the sliding speed on the tribological properties of the three composite. With the increase of sliding speed, the friction coefficient reduced, and the wear rate increased of the composites, mainly because of the increase of the sliding speed increases the frictional heat in the sliding process, causing friction surface matrix softening, friction force is reduced, the fiber and matrix more easily fall off from composite materials.^41^ The friction coefficient and wear rate of GO–PDA–BF/PA6 composites are always at the lowest value compared with the other two composites. This imply that the improvement of interfacial bonding strength is the main factor to improve the carrying capacity and the wear-resisting property of the composites.

#### 3.3.4. Morphology of the wear surface and wear debris

**Fig. 11:**
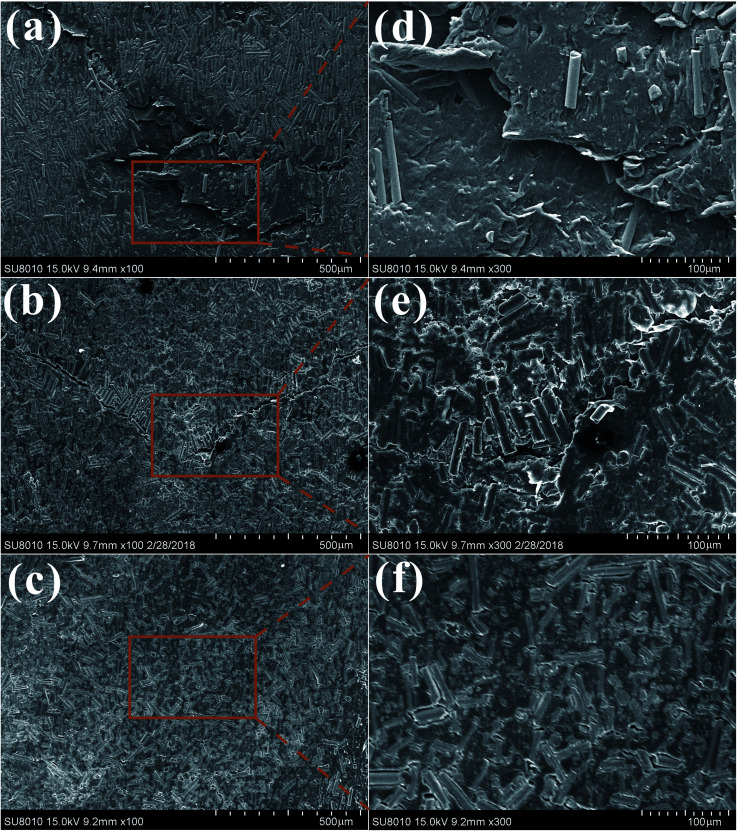
SEM images of worn surfaces of BF/PA6 (a), PDA–BF/PA6 (b), GO–PDA–BF/PA6 (c) and their corresponding magnified images marked as (d), (e) and (f), respectively.

**Fig. 12:**
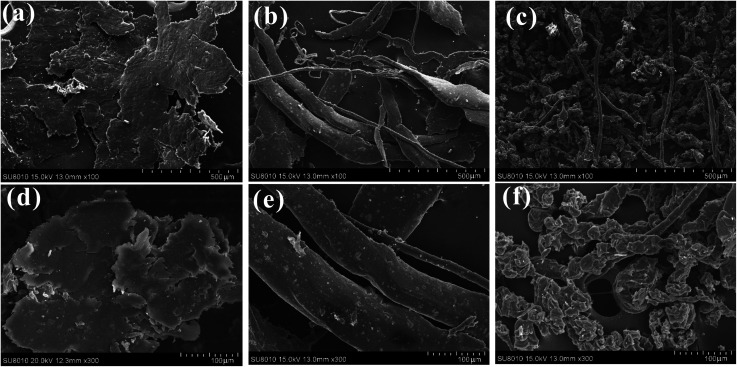
SEM images of wear debris: (a) BF/PA6, (b) PDA–BF/PA6, (c) GO–PDA–BF/PA6 and their corresponding magnified images marked as (d), (e) and (f), respectively.

SEM images of worn surfaces and wear debris for the composites were shown in Fig. 11 and 12. For BF/PA6 composite, the wear surface is badly damaged and the composite in the worn area breaks, lots of fiber and matrix were shelled off (see Fig. 11a and d and 12a and d) owing to its poor interfacial properties. With regard to PDA–BF/PA6, with the increase of interfacial bond strength, the damage degree of the worn surface has been effectively suppressed, fewer fibers debond from the matrix. Compared with BF/PA6 composite, the crack on the worn surface decreases obviously and the wear debris decreases as well (see Fig. 11b and e and 12b and e). As shown in Fig. 11c and f and 12c and f, the worn surface is smooth without obvious damage and fiber debonding phenomenon, and the debris is transformed from the massive sheet of BF/PA6 composite to the small particles of GO–PDA–BF/PA6 composite. This is the result of the improvement of the properties of the interface between the fiber and the matrix and the excellent lubrication and wear reduction of graphene oxide.

**Fig. 13:**
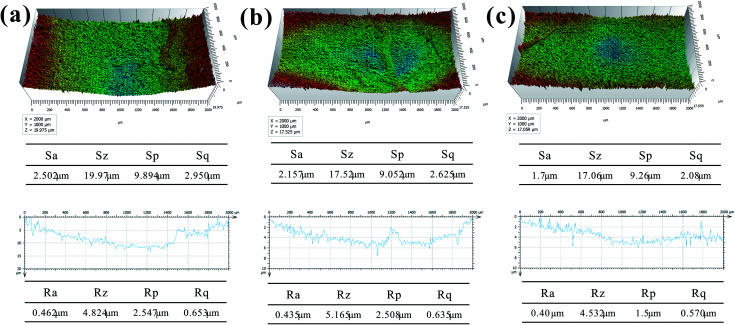
3D topographies of worn surface: (a) BF/PA6, (b) PDA–BF/PA6 and (c) GO–PDA–BF/PA6.

**Fig. 14:**
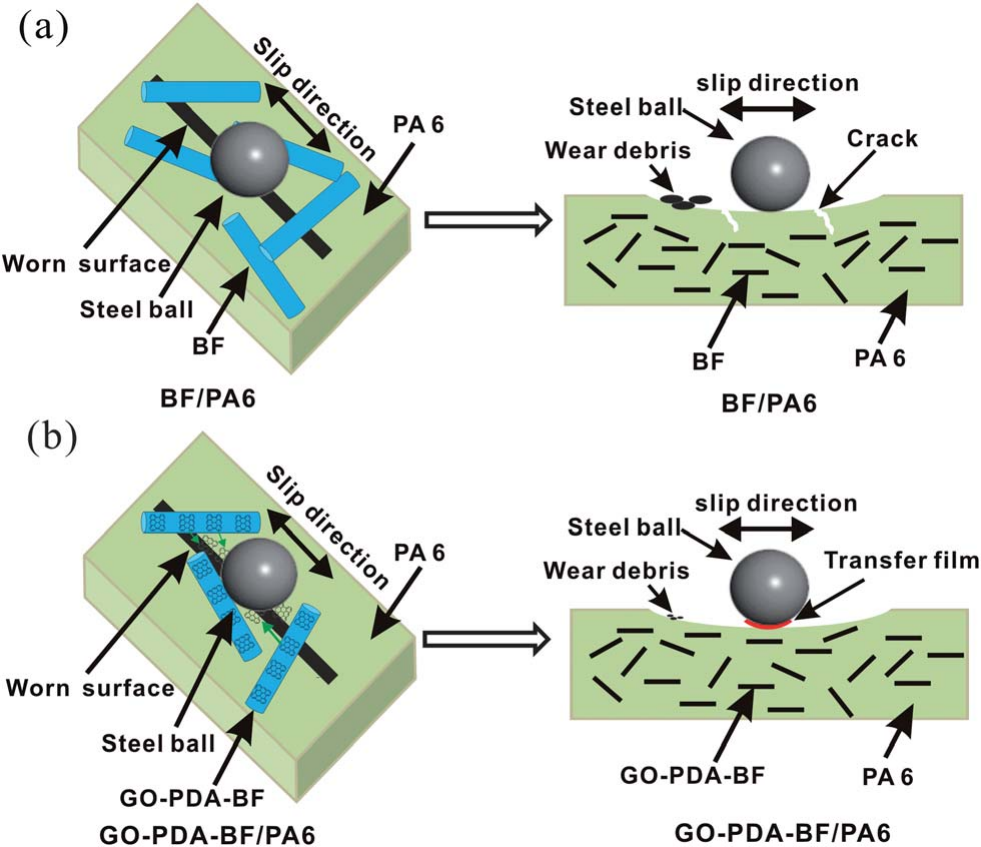
Schematic representation of wear mechanism: (a) BF/PA6 and (b) GO–PDA–BF/PA6.

Roughness and morphology of wear surface of BF/PA6, PDA–BF/PA6 and GO–PDA–BF/PA6 composites at 70 N and 0.167 m s^−1^ were characterized by a 3D non-contact surface topography instrument in which Fig. 13a–c are the wear surface of composites before and after fiber modification, respectively. The surface roughness parameters (*S*_a_, *S*_p_, *S*_z_, and *S*_q_) presented in the tables in Fig. 14, show that the wear depth and surface roughness of the PDA–BF/PA6 and GO–PDA–BF/PA6 composites are lower than BF/PA6 composites. The surface roughness of PDA–BF/PA6 composite decreased slightly, while that of GO–PDA–BF/PA6 composite decreased significantly, thus the GO grafting onto fiber surface could effectively reduce the surface roughness of wear surface.^42^

#### 3.3.5. Analysis of wear mechanism

Based on the above considerations, the friction mechanism of fiber reinforced composites in the process of friction can be proposed before and after modification. Fig. 14a shows the mechanism of BF/PA6 composites in the process of friction. As it is known that the hardness of steel ball is much harder than PA6 composites. Therefore, under the load stress and the sliding speed, the projecting part of the steel ball will enter the surface of the composites, and the softened matrix is stripped out to form the wear surface. Under the cyclic stress in the process of friction, due to the poor interfacial adhesion between fiber and matrix in BF/PA6 composites, part of BF and PA6 will peel off along with the softer matrix and become wear debris, and easy to form damage on worn surfaces, as is confirmed in Fig. 11a and d and 12a and d. Fig. 14b presented the schematic of GO–PDA–BF/PA6 composites during the friction process. Due to the strong interface bonding strength and anti-wear protective transfer film formed by the peeling GO on the worn surface of GO–PDA–BF/PA6 composites, the cracks initiation and growth will be hindered, the debonding of BF and matrix damage will also be alleviated, thus the wear of composites can be inhabited and the consequently wear debris became smaller, which can be seen in Fig. 11c and f and 12c and f. Besides, under the action of cyclic stress, GO with high ductility and flexibility is more easily deformed which can effectively buffer the stress, inhibit the generation and expansion of cracks, thus reducing the friction coefficient and improving wear resistance of composites.^43^

## 4. Conclusions

In summary, a graphene oxide layer was immobilized covalently onto basalt fiber surfaces by bioinspired polydopamine coating and graphene oxide grafting under a simple and gentle method. A large number of active functional groups have been introduced onto the fiber surface and the roughness of fiber surface has been increased, which improves the chemical coupling and mechanical meshing between basalt fiber and PA6 matrix. In consequence, the GO–PDA–BF/PA6 composite increased by 13.6% and 12.7% in impact and flexural strength, respectively, as compared to BF/PA6 composite. Furthermore, the friction and wear tests showed the wear rate of GO–PDA–BF/PA6 composite decreased by 51% compared with BF/PA6 composites and it also displayed the optimal anti-wear and load-carrying ability under various applied load and sliding speed, which can be explained by the improved interface bonding between GO–PDA–BF and PA6 matrix and anti-wear protective transfer film formed by GO. Most importantly, the study provides a simple and efficient method that has no damage to the materials. It can introduce various nanomaterials onto various substrates according to the different uses of composites and achieve the directional modification of composites through the combination of dopamine coating and chemical grafting.

## Conflicts of interest

There are no conflicts to declare.

## Supplementary Material
